# Supplementary material for: Polydatin-Induced Shift of Redox Balance and Its Anti-Cancer Impact on Human Osteosarcoma Cells
Source: Curr Issues Mol Biol. 2024 Dec 31;47(1):21. doi: 10.3390/cimb47010021 (PMC11764470; doi:10.3390/cimb47010021)
Supplement: Supplementary file 1 [file cimb-47-00021-s001.zip › cimb-3399614-supplementary.pdf]

Supplementary Materials

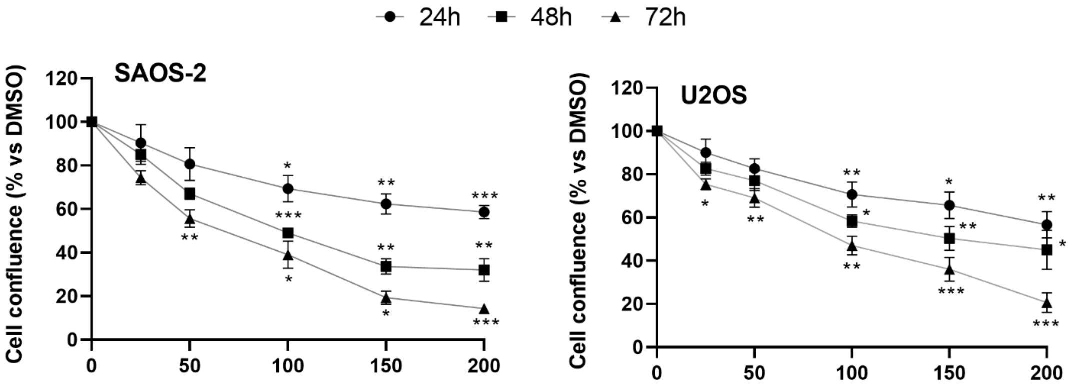

Supplementary Figure S1: Proliferation assay on uncoated plates of SAOS-2 and U2OS cells following PD treatment. The proliferation of SAOS-2 and U2OS cells was evaluated by measuring cell live confluence after exposure to PD (25–200 µM) for the indicated time points, using the confluence function of the Spark microplate reader (Tecan Group). Cell confluence was calculated as a percentage by normalizing the values from PD-treated cells to those of DMSO control cultures. Data are presented as the mean ± SD from three independent experiments, each performed in triplicate (*p < 0.05, **p < 0.01, ***P<0.001 vs DMSO).

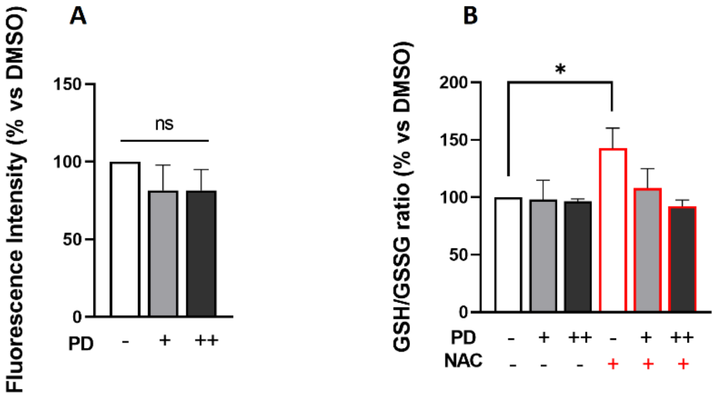

Supplementary Figure S2: Measurement of ROS and GSH levels in hFOB 1.19 cells. (A) DCF fluorescence assay. Cells were treated with PD 100 µM (+) or 200 µM (++) for 24 h. ROS are expressed as percentage of DCFDA fluorescence intensity compared to DMSO-treated cells arbitrarily set at 100%. **(B)** Cellular GSH/GSSG ratio measured by fluorometric microplate format. hFOB 1.19 cells were treated with PD 100 µM (+) or 200 µM (++) for 24 h, with or without 1mM NAC, 1h prior to PD treatment. Ratios are reported as percentage change compared to vehicle control. The results are shown as means ± SD from three

independent experiments performed in triplicate (*P < 0.05 compared with control group). 'ns' indicates that the difference is not statistically significant.

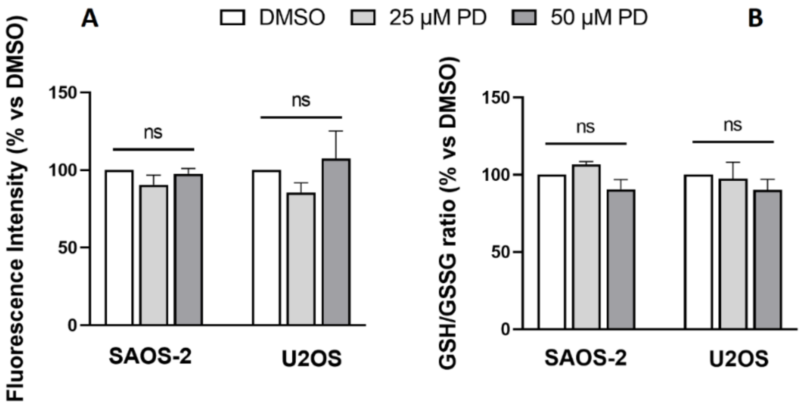

Supplementary Figure S3: SAOS-2 and U2OS cells were treated with PD 25 µM and 50 µM for 24 h. **(A)** ROS are expressed as percentage of DCFDA fluorescence intensity compared to DMSO-treated cells arbitrarily set at 100%. Results are given as the mean ± SD of three independent experiments performed in triplicate. **(B)** GSH/GSSG ratio measured by fluorometric assays. Ratios are reported as percentage change compared to vehicle control. Data are presented as the mean ± SD of three independent experiments with triplicate sets in each assay. 'ns' indicates that the difference is not statistically significant.

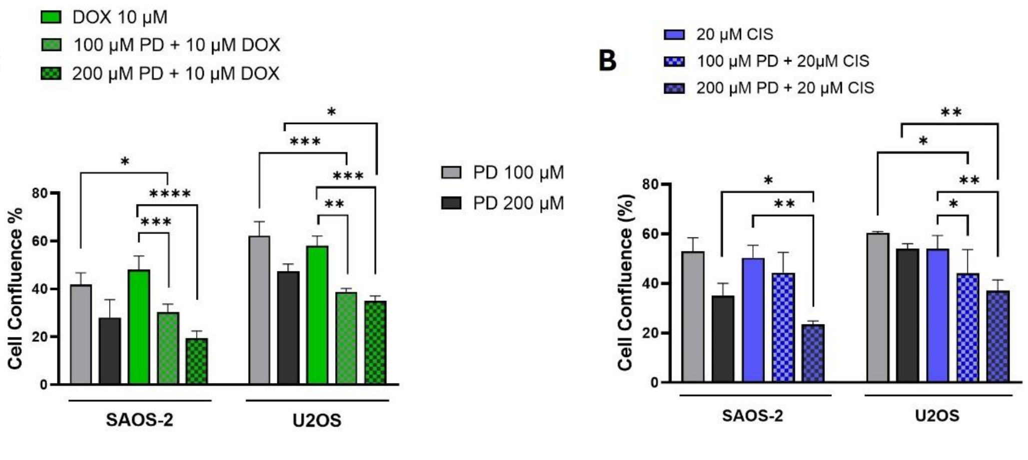

Supplementary Figure S4: Proliferation assay on non-coated plates of SAOS-2 and U2OS cells treated with PD in combination with DOX (A) and CIS (B). Cell confluence was recorded with the live imaging program of a TECAN sparkR multimode reader. The data are reported as percentage values of cell confluence for cells treated with PD, DOX/CIS, or a combination of DOX/CIS and PD, relative to the untreated control group (set to 100%) after 48 hours of treatment. Values represent mean ± SD of three independent experiments.

Values represent mean ± SD of three independent experiments (*$p < 0.05$, **$p < 0.01$, ***$p < 0.001$, ****$p < 0.0001$).
